# A Pilot Study Assessing the Impact of a Fortified Supplementary Food on the Health and Well-Being of Crèche Children and Adult TB Patients in South Africa

**DOI:** 10.1371/journal.pone.0055544

**Published:** 2013-01-30

**Authors:** Michael Rudolph, Florian Kroll, Moira Beery, Edmore Marinda, Jean-Francois Sobiecki, Geoffrey Douglas, Gary Orr

**Affiliations:** 1 Siyakhana Initiative, University of the Witwatersrand Health Consortium, Johannesburg, Gauteng, South Africa; 2 School of Public Health, University of the Witwatersrand Faculty of Health Sciences, Johannesburg, Gauteng, South Africa; 3 Centre for Anthropological Research, Faculty of Humanities, University of Johannesburg, Gauteng, South Africa; 4 Health Empowerment Through Nutrition, Malvern, Worcestershire, United Kingdom; University of Ottawa, Canada

## Abstract

The South African population faces many of the global concerns relating to micronutrient deficiency and the impact this has on health and well-being. Moreover, there is a high prevalence of HIV infection, compounded by a high level of co-infection with TB.

This pilot study evaluates the impact of a fortified supplementary food on the health and well-being of a cohort of crèche children, aged 3 to 6, and adult TB patients drawn from the Presidential Node of Alexandra, Johannesburg, South Africa. A further aim of this study was to evaluate the sensitivity and validity of non-invasive indicators of nutritional status in a field-based population sample.

The investigational product, e’Pap, is supported by extensive anecdotal evidence that whole grain cereals with food-style nutrients constitute an effective supplementary food for those suffering from the effects of food insecurity, poor health and well-being, and coping with TB and HIV infection.

The results indicate a beneficial effect of e’Pap for both study populations, and particularly for adult TB patients, whose baseline data reflected severe food insecurity and malnutrition in the majority of cases. There is evidence to suggest statistically significant improvements in key micronutrient levels, well-being and energy, hand-grip strength, the Bioelectrical Impedance Analysis (BIA) Illness Marker, and certain clinical indicators. Although Body Mass Index (BMI) and Mid Upper Arm Circumference (MUAC) are frequently used as standard measures to evaluate the efficacy of nutritional interventions, these indicators were not sufficiently sensitive in this study. Nor does weight gain necessarily indicate improved nutritional status. Hand-grip strength, lean body mass, and the BIA Illness Marker seem to be more useful indicators of change in nutritional status.

## Introduction

Undernutrition is a serious but least addressed global health problem [1]. Undernutrition interacts with repeated bouts of infectious disease, causing an estimated 3.5 million preventable maternal and child deaths annually [2]. Addressing undernutrition is essential to meeting the Millennium Development Goals (MDGs) [3].

Micronutrient deficiencies are common in South Africa, where the majority of the population depends on a diet of refined maize and wheat flour products [4]. Highly refined cereal flours lose a large part of their nutrients during processing [5]. Consequent negative outcomes include lower energy levels, lower educational attainment, impaired immunity and higher rates of disability and illness [6]. Those at highest risk are children, pregnant and breastfeeding mothers and immune compromised people, such as those living with HIV/AIDS and/or TB [7]. Nutritional deficiencies of vitamin B12, zinc and selenium in malnourished people living with HIV/AIDS are associated with decreased immunity and a higher risk of disease progression [8], [9]. Problems related to childhood malnutrition and micronutrient deficiency include stunting of growth, low body weight, decreased physical activity and reduced resistance to disease [10].

Reduced micronutrient intake (most noticeably vitamins A, C & E and selenium & zinc) has been associated with impaired immunity [11]–[15]. The association between TB and malnutrition has long been known [15]–[26]. Malnutrition weakens immunity, increasing the chance that latent TB will develop into active disease. Where TB patients receive Vitamins A, B-complex, C, E and selenium, there is a reduction in neuropathy and a reduction in recurrence [16]–[25]. In healthy people who have been exposed to tuberculosis, a single oral dose of vitamin D enhances their immunity to infection [26]. Nutrition interventions have improved outcomes, including susceptibility to opportunistic infections and response to medical treatment [27].

TB and HIV significantly compromise the food security of affected communities, reducing the availability of productive labour, diverting income, depleting savings and productive assets, overwhelming social networks and safety nets, and impeding intergenerational knowledge transfers [26]. Yet, little is known about effective nutritional management. A study panel from the Academy of Science of South Africa has noted the dearth of reliable studies on the influence of nutritional interventions on the course and outcomes of pandemic chronic diseases, such as HIV/AIDS and TB [28].

In response to the findings of the National Food Consumption Survey [4], [29], the South African Department of Health introduced mandatory food fortification, which, since October 2003, has required the addition of iron, zinc, vitamin A, thiamine, riboflavin and vitamin B6 to all maize and wheat flour. Vitamin and mineral enriched foods should be vital for individuals suffering from illnesses such as TB or HIV, but there is little evidence demonstrating their efficacy and effectiveness. Iron and zinc fortification, using inorganic salts, has shown little impact on plasma levels, and most studies investigating food fortification programmes across Africa, using chemical isolates, show little positive impact on health [30]. Indeed, a recent Cochrane Review [31] states, ‘Although the impact of supplementary feeding on child growth appeared to be negligible, it is not possible to draw any conclusions until we have studies that involve larger numbers and do not allow assessors to know who is receiving the intervention.’

The Lancet defines thirteen cost-effective interventions to address maternal and child undernutrition [1]. Nine of these involve micronutrient supplementation. However, there remains limited knowledge about the bioavailability and bioefficacy of such interventions.

Health Empowerment Through Nutrition (HETN) had been working with the South African National Tuberculosis Association (SANTA)-adult TB patients-and the Society of St Vincent de Paul (SSVP)-crèche children-for over 18 months, providing supplementary feeding with the investigational product, e’Pap. The unanimous feedback was that the intervention was transforming lives. SANTA reported improvements in well-being and TB cure rates. SSVP reported that children who had previously slept all day were now alert and interacting. Despite the clear benefit witnessed by observers, their evidence remained anecdotal. Both SANTA and SSVP were keen to quantify their observations, so the University of the Witwatersrand (WITS) was approached to conduct a pilot study. Although the two study populations are different, the same methodology was applied to each, and it was felt appropriate for a pilot study to produce a single report covering both populations.

Traditional and recent nutrition interventions use refined cereals or high fat and sugar pastes, fortified with vitamins and minerals in the form of chemical isolates. The hypothesis, based on extensive anecdotal evidence, is that whole grain cereals with food-style nutrients constitute a more effective intervention. The aims of this 3-month pilot study with crèche children and adult TB patients in Alexandra, South Africa, were to generate baseline data on nutritional status, assess the impact of a fortified supplementary food (e’Pap) on nutritional status, and to evaluate the sensitivity and validity of non-invasive indicators of nutritional status. This paper is an overview of the pilot study, highlighting the range of instruments used to assess nutritional status and reporting on some of the main findings.

## Methods

### Ethics Statement

Ethics approval was granted by the Faculty of Health Science’s Ethics Committee at the University of the Witwatersrand (WITS), Johannesburg, South Africa. Community health workers, employed by the South African National Tuberculosis Association (SANTA), approached participants for their consent. Informed written consent was obtained from all participants or their guardians.

### Sample and Sampling

This study was conducted in Alexandra, in north eastern Johannesburg, South Africa. Alexandra, a Presidential Node, has been identified by the SA Government as an area of exceptional need. Over half a million people live in an area of approximately two square kilometres. HIV/AIDS, TB and malnutrition are rife. There are many orphans and vulnerable children. As this population is defined as high need, and low micronutrient status had been shown by the National Food Consumption Survey [4], it was decided not to select subjects based on a pre-determined malnutrition assessment. Two cohorts were selected that were accessible via clinics and crèches in the vicinity of the community centre that was made available for the study.

The Kholofela ya Joseph Community Centre in Alexandra served as the headquarters for the study. This Centre is run by SSVP and is also the site of SANTA’s office in Alexandra. Child data collection took place at St Martin’s Preschool and Thabisong Preschool in Alexandra. Adult data collection took place at the East Bank Municipal Hall.

The adult cohort was drawn from persons satisfying the following criteria:

Inclusion: male and female outpatients, between the ages of 18–60; receiving TB treatment from a clinic in Alexandra; a history of at least 3 regular clinic visits; BMI≥16; consenting to participate in the study for its full duration. Exclusion: consuming e'Pap or a similar fortified supplementary food; allergy to maize or soya; receiving treatment for Multidrug-Resistant TB.

The child cohort was drawn from persons satisfying the following criteria:

Inclusion: male and female children, between the ages of 3–6; attending informal crèches in Alexandra; a history of regular crèche attendance; MUAC≥110 mm; written consent to participation from the child’s parent or legal guardian. Exclusion: consuming e'Pap or a similar fortified supplementary food; allergy to maize or soya.

Intervention and data collection took place from September-December 2010. 87 adult TB patients and 68 crèche children were recruited. All participants were de-wormed at baseline, using a single dose of 100mg Mebendazole, to ensure that nutrition was not compromised by parasitic infestation.

Participants were supplied with and were requested to consume the nutrition intervention, e’Pap (adults 100 g, children 50 g) daily, mixed with cool or warm water, or sprinkled over cooked food, and return for 2 subsequent data collections, 1 month apart. Adult participants, who ate the product at home, were interviewed about compliance. The great majority reported adherence to the recommended use. For child participants, the supplement was served twice daily at the crèches, where consumption adhered closely to what was recommended. Supplies were taken home along with instructions for weekend feeding.

### Assessment Tools

A Nutritional Indicator Survey Tool was used to record socio-economic profile, self-reported HIV status, self-reported general well-being (1 = very bad, 2 = bad, 3 = OK, 4 = good, 5 = very good), household size and employment status.

The participants were examined for signs of malnutrition in the hair, eyes, lips, gums, tongue, skin and nails.

The Household Food Insecurity Access Scale (HFIAS) [32] and a 24-hour Individual Dietary Diversity Score (IDDS), based on the Household Dietary Diversity Score (HDDS) [33], were used to assess food security and the diversity of foods consumed by participants. The HFIAS is a standardised questionnaire consisting of 9 questions which measure different aspects of food access insecurity, with additional probing as to the frequency of occurrence. The responses are coded in terms of the frequency of occurrence and are then summed to arrive at the score. The HFIAS score is a continuous measure of the degree of food insecurity in the household in the past 30 days. The higher the score (ranging from 0 to 27), the more food insecure the household is considered to be.

Height (cm) was measured using a Seca Mobile Stadiometer 214. Weight (kg) was measured using a Seca Sensa 872 scale. Waist and hip circumference (cm) were measured using a standard sewing tape. Mid-upper arm circumference–MUAC (cm) was measured in children using a MUAC tape.

Bioelectrical Impedance Analysis (BIA) was performed using a Bodystat Quadscan 4000, from which it is possible to derive Lean Body Mass (LBM) and the Illness Marker (IM) [34], which is thought to reflect the integrity of cell membranes. The Illness Marker is the ratio between the impedance measurement at 200 kHz and at 5 kHz. At 200 kHz, the current is strong enough to penetrate the cell membrane and therefore total body water (TBW) is measured. However, at 5 kHz, the membrane cannot be penetrated and only extracellular water (ECW) is measured. These figures are expressed as a ratio. The greater the variance between the two impedance values, the healthier the body. A ratio close to 1.00 indicates poor cellular health or extreme fluid overload. Expansion of ECW and loss of ICW are typical features of systemic illness, arising from a loss of intracellular protein which leaks into the extracellular space. Participants were not advised to drink prior to testing.

Hand-Grip Strength was measured using a Takei GRIP-D Grip Strength Dynamometer.

10ml of venous blood was collected from all participants at each data collection. Participants were not advised to fast. Serum levels of zinc (flame atomic absorption spectroscopy), iron (ferrozine assay), selenium (graphite furnace atomic absorption spectroscopy), vitamin A (high performance liquid chromatography with UV detector-HPLC-UV), vitamin D–25(OH)D (HPLC-UV) and albumin (BCG dye binding) were measured by the National Health Laboratory Service (NHLS) at WITS.

Seven community health workers, employed by SANTA, were trained to administer the questionnaires, measure height, weight and hand-grip strength, and to record the BIA. This training was provided by nurses from the WITS Medical School, using standardised procedures, and it was repeated twice before the data collection began. Each health worker dealt with a different indicator. Blood samples, observation of the clinical signs of malnutrition, and waist and hip circumference were measured by experienced nurses, using standardised procedures.

### Investigational Product-Table 1

**Table 1:** e’Pap Composition.

	Unit	Per 100 gm	Adult RDA	% of Adult RDA per 100 gm
Energy	KJ	1556		
Protein	gm	12.7		
Carbohydrate	gm	63.6		
Total Cereal Fat	gm	7		
Total Dietary Fibre	gm	10		
Potassium	%	0.46		
Sodium	%	0.5		
Vitamin A	RE	1000	1000	100
Vitamin B1	mg	1.4	1.4	100
Vitamin B2	mg	1.6	1.6	100
Vitamin B3	mg	18	18	100
Vitamin B5	mg	6	6	100
Vitamin B6	mg	2	2	100
Vitamin B12	µg	1	1	100
Vitamin C	mg	60	60	100
Vitamin D3	µg	5	5	100
Vitamin E	mg	10	10	100
Folic Acid	µg	200	200	100
Biotin	µg	100	100	100
Iron	mg	14	14	100
Zinc	mg	15	15	100
Iodine	µg	150	150	100
Calcium	mg	220	800	28
Magnesium	mg	45	300	15
Manganese	mg	0.45		
Copper	mg	0.3		
Selenium	µg	200		
Vanadium	µg	50		
Chromium	µg	30		
Molybdenum	µg	30		

A fortified supplementary food called e’Pap was chosen for this pilot study because it is whole grain, unlike many supplementary foods that are based on refined cereal flours which lose a large part of their nutrients during processing [4]. e’Pap uses chelates, unlike many supplementary foods that are fortified with minerals as chemical isolates. Phytic acid in cereals has a strong binding affinity to important minerals, such as calcium, magnesium, iron and zinc. When a mineral binds to phytic acid, it becomes insoluble and non-absorbable. This can contribute to mineral deficiencies in people whose diets rely on these foods for their mineral intake [35], [36]. Finally, e’Pap is pre-cooked, unlike many supplementary foods that require cooking, and cooking destroys vitamins. It is also tasty.

### Statistical methods

To describe micronutrient levels among children and adults, means (standard deviations) and medians (inter-quartile range Q1–Q3) were used, while means (standard deviations) were used to describe anthropometric measurements (height, weight and BMI) among all participants. Frequencies (percentages) were used to describe categorical variables such as sex, HIV status and employment status. Where comparisons were made, Chi-squared tests (or Fisher’s exact test), t-tests (Mann-Whitney test) or Analysis of Variance (ANOVA, Kruskal-Wallis test) were used as appropriate. In order to assess changes in micronutrient levels over time, we used linear regression models. The two follow-up micronutrient measurements were compared with baseline levels accounting for multiple measurements for each individual. We combined adult and child data, and adjusted for age in order to gain on sample size. All statistical tests were considered statistically significant at 5%, and regression estimates were reported with 95% confidence intervals. STATA version 11 (StataCorp, College Station, USA) was used for all statistical analysis.

## Results

A total of 87 adult TB patients-50 females (57%) and 37 males (43%)-and 66 children–33 males (50%) and 33 females (50%)-were recruited. Adult females were younger (mean age 34 years, STD 10 years) than males (mean age 40 years, STD 9 years). The mean age for female children was 4.6 years (STD 0.8 years) and for male children 4.7 years (STD 0.9 years). The median household size was 4 (Q1–Q3 3–4). Unemployment was high among recruited adult participants-69% among males and 59% among females.

Table 2 shows the losses to follow up. 6 adult and 2 child participants withdrew from the study, reporting complications, which included rashes, mouth sores and vomiting. As many of these issues in the adult participants are also effects of HIV, and could be linked to the side-effects of medication, it was not possible to link them to any particular cause, including consumption of the supplement. Other reasons for adults defaulting were inability to communicate with and inform participants of data collections days, schedule conflicts with daytime data collections, lack of time available to spend at lengthy data collections, which could take 1–4 hours, admission to hospital, dislike of invasive tests and dislike of the taste of e’Pap. Other reasons for child loss to follow up were dislike of invasive tests and early withdrawal from school at the end of the school year.

**Table 2:** Losses to Follow Up.

	Baseline	Visit 1	Visit 2
**Adult Male**	37	19	12
**Adult Female**	50	39	27
**Adult Total**	**87**	**58**	**39**
**Child Male**	33	32	30
**Child Female**	33	31	27
**Child Total**	**64**	**63**	**57**

### Health Status

67% of the adult TB patients (76% of females; 54% of males) self-reported as being HIV positive. There were no reports of HIV positive serology in the child cohort. There was a high prevalence of oral candidiasis among adult TB patients (35%) which indicates compromised immunity.

**Figure 1:**
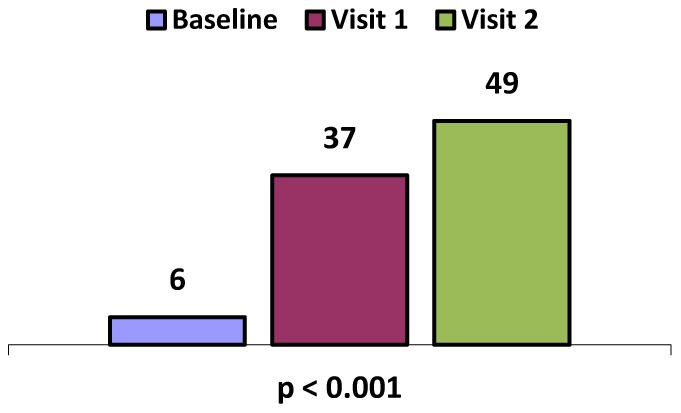
Adult % General Well Being.

Among adult participants, only 6% reported feeling good or very good at baseline. This increased significantly (p<0.001) to 37% at Visit 1 and 49% at Visit 2, as shown in Figure 1. This was associated with improvements in the clinical indicators of malnutrition. In contrast, children’s' feelings of well-being remained high throughout (91% to 93%).

### Clinical Indicators

A number of clinical indicators of malnutrition can be assessed visually, particularly those manifesting in the skin [37].

Trained nursing staff examined the participants for signs of malnutrition in the hair (lack of shine, thin, sparse, loose, flag sign), eyes (dry, foamy spots, night blindness, redness), lips (cheilosis, angular stomatitis), gums (spongy, bleeding), tongue (sore, smooth), skin (dry, scaling) and nails (spoon shaped, brittle, ridged, pale). Participants were advised to refrain from using skin lotions or hair conditioners for 24 hours prior to the examinations.

Statistically significant improvements were observed in the clinical signs of malnutrition in the eyes, from 50% at baseline to 24% after 3 months (p = 0.01), and nails, from 50% at baseline to 26% after 3 months (p = 0.04). Although changes in the other clinical malnutrition markers did not reach statistical significance, they all showed some improvement.

### Food Security and Food Diversity

Nearly all adults (97% of females and 96% of males) reported being food insecure. Food insecurity was also relatively high (57%) among children-as reported by parents/ guardians. Reported levels of food security did not change over the course of the study.

Mean dietary diversity scores for adults were below the minimum target of 7 out of 12 (adult males 6.1; adult females 5.0; child males 7.3; child females 7.5). Diets-particularly among adults-reflected a strong reliance on starchy staples (refined maize porridge and bread), with a lower consumption of protein, fruit and vegetables.

### Anthropometry

Table 3 and Table 4 show the anthropometric measurements of adults and children. The mean baseline BMI among adult males was low (19.2), while in adult females it was normal (23.3).

**Table 3:** Adult Anthropometry.

	Adult Males-Mean (STD)			Adult Females-Mean (STD)		
	Baseline	Visit 1	Visit 2	Baseline	Visit 1	Visit 2
Weight (kg)	57.3 (7.8)	60.4 (9.1)	57.9 (10.3)	61.3 (15.2)	61.9 (15.0)	63.2 (15.4)
Height (cm)	170 (20)	167 (27)	161 (32)	158 (14)	160 (8)	161 (8)
BMI (kg/m^2)^	19.2 (2.0)	19.8 (2.3)	20.0 (2.2)	23.8 (5.0)	24.2 (5.1)	24.4 (5.2)
Hip (cm)	83.9 (7.2)	88.6 (7.7)	85.7 (5.2)	95.1 (14.2)	96.7 (11.9)	96.8 (11.4)
Waist (cm)	75.4 (5.3)	76.6 (5.8)	76.6 (6.7)	80.1 (10.8)	80.0 (9.7)	82.2 (9.5)
Waist/Hip Ratio	0.90 (0.16)	0.87 (0.1)	0.89 (0.06)	0.84 (0.09)	0.84 (0.07)	0.85 (0.10)

Note: BMI = Body Mass Index

**Table 4:** Child Anthropometry.

	Male Children-Mean (STD)			Female Children-Mean (STD)		
	Baseline	Visit 1	Visit 2	Baseline	Visit 1	Visit 2
Weight (kg)	18.2 (3.4)	18.6 (3.3)	18.6 (3.3)	18.2 (3.9)	18.6 (3.9)	19.6 (5.8)
Height (cm)	107 (78)	107 (7)	107 (7)	107 (8.5)	108 (7)	107 (7.3)
BMI (kg/m^2)^	15.6 (1.5)	16.1(1.6)	16.0 (1.5)	15.8 (2.0)	16.2 (2.3)	17.3 (6.4)
Hip (cm)	55.0 (5.2)	57.9 (4.8)	57.6 (5.1)	57.1 (7.8)	59.8 (6.4)	58.7 (5.8)
Waist (cm)	51.2 (4.2)	52.8 (6.9)	51.8 (4.2)	52.5 (4.5)	52.4 (4.7)	49.7 (10.5)
Waist/Hip Ratio	0.93 (0.05)	0.91 (0.07)	0.90 (0.04)	0.92 (0.08)	0.88 (0.04)	0.84 (0.18)
MUAC (cm)	16.9 (1.6)	17.0 (1.5)	16.8 (1.4)	17.2 (1.8)	17.2 (1.8)	17.1 (1.99)

Note: BMI = Body Mass Index; MUAC = Mid Upper Arm Circumference

**Figure 2:**
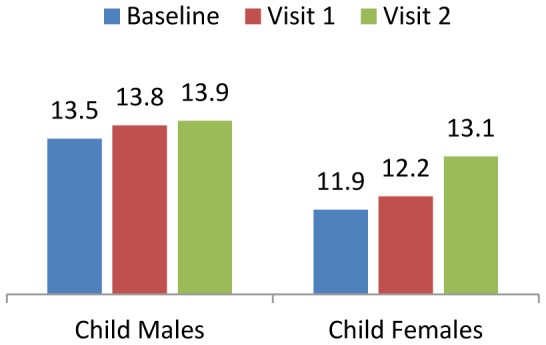
Child Lean Body Mass.

All groups exhibited an increase in BMI, while waist to hip ratios remained stable. Child baseline median body weight was 18.1kg for girls and 17.2kg for boys, compared to WHO international growth standards of 18.2kg for girls and 18.3kg for boys. Lean body mass, as measured by bioelectrical impedance, showed a consistent increase in the child cohort, as shown in Figure 2. It increased on average by 1.9% (p = 0.01) from baseline to visit 1, and increased by 2.5% (p = 0.01) from baseline to visit 2.

**Figure 3:**
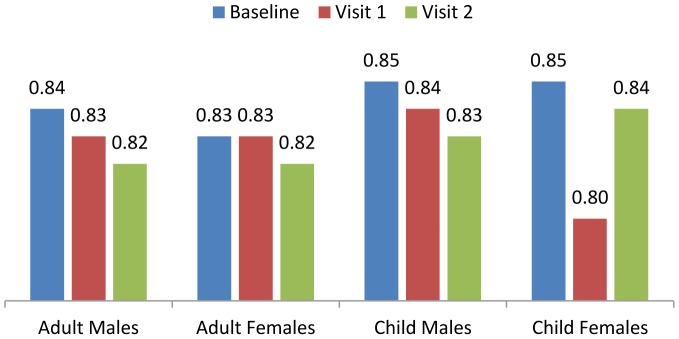
Illness Marker.

Bioelectrical Impedance Analysis (BIA) reflected a slight but consistent improvement of the Illness Marker among adults and children, as shown in Figure 3. HIV negative adults showed greater improvement (mean change of 0.013, 95% CI -0.020-0.006) over three months than HIV positive adults (mean change of 0.007, 95% CI -0.021-0.082). Baseline levels for both cohorts were far above the reference value of 0.75 for healthy populations, and were actually closer to levels observed among critically ill subjects [34].

**Figure 4:**
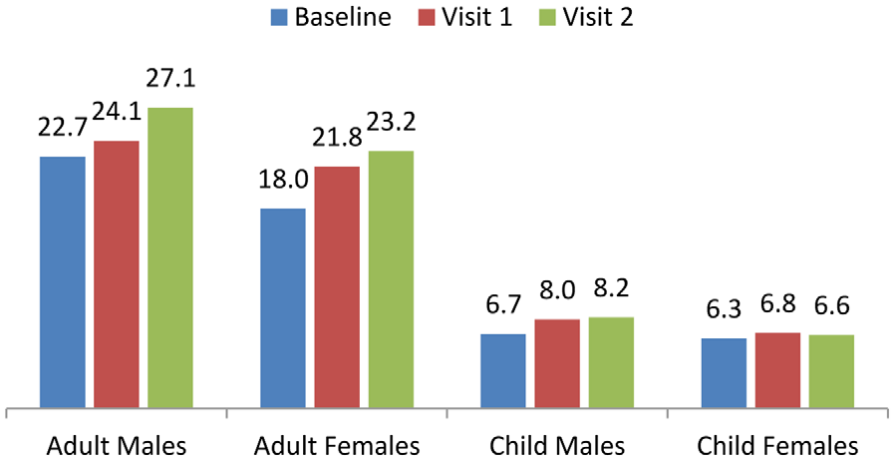
Mean Hand-grip Strength.

Mean hand-grip strength improved in both adult and child cohorts, with significant changes (p = <0.01) among female adults, with values of 18, 22 and 23 at baseline, Visit 1 and Visit 2, and among child males (p = 0.01) with values of 6.7, 8 and 8.2 at similar visits. These results are displayed in Figure 4. In adults, this change mirrored reported improvement in general well being and energy.

### Biochemistry

Initially, the intent was to collect data that would enable conclusions to be reached about longitudinal change over time as a result of supplementation. Due to budgetary constraints, blood sample analysis in the last assessment was prioritised for participants who had attended all three assessments. Laboratory related complications in labelling and storage further reduced the number of analysed samples that could be included in the study by approximately 25%.

Table 5 and Table 6 show the changes in serum biochemistry among adult TB participants and child participants respectively. Approximately half the adult participants had levels of iron, zinc, albumin, vitamin A, and vitamin D below the international normal range, indicating clear micronutrient deficiency. The child population’s diet at baseline included some fruits, vegetables and protein each day, yet blood analysis showed that 32%, 40% and 6% of them had selenium, iron and zinc levels respectively below the normal range.

**Table 5:** Adult Biochemistry.

	Adult Males			Adult Females		
	Baseline	Visit 1	Visit 2	Baseline	Visit 1	Visit 2
**Selenium (µg/L)**	n = 23	n = 11	n = 8	n = 21	n = 13	n = 19
Median (Q1–Q3)	62 (56–72)	71 (58–83)	82 (55–86)	68 (61–86)	52 (50–68)	68 (51–80)
Mean (STD)	64 (15)	68 (21)	76 (22)	71 (19)	58 (14)	66 (16)
**Albumin (g/L)**	n = 27	n = 14	n = 10	n = 28	n = 19	n = 24
Median (Q1–Q3)	36 (30–39)	38 (36–40)	36 (35–40)	38 (34–41)	39 (50–68)	39 (35–42)
Mean (STD)	35 (5)	38 (5)	37 (4)	38 (5)	39 (4)	38 (4)
**Iron (mmol/L)**	n = 27	n = 14	n = 10	n = 28	n = 19	n = 24
Median (Q1–Q3)	10.8 (7.1–18.1)	12.2(8.1–18.8)	13.3(9.8–18.9)	8.7 (5–14.0)	11.8(7.2–15.2)	9.2 (6.7–14.0)
Mean (STD)	12.3 (6.7)	13.7 (5.8)	14.1 (4.9)	10.4 (6.7)	11.3 (5.1)	10.8 (6.4)
**Vit A (mmol/L)**	n = 26	n = 14	n = 10	n = 26	n = 19	n = 23
Median (Q1–Q3)	0.97 (0.65–1.36)	0.99 (0.76–1.26)	1.04 (0.96–1.37)	1.08 (0.87–1.41)	1.14 (0.94–1.46)	1.14 (0.83–1.58)
Mean (STD)	1.04 (0.45)	1.05 (0.38)	1.09 (0.34)	1.15 (0.43)	1.20 (0.37)	1.22 (0.52)
**Vit D (nmol/L)**	n = 24	n = 9	n = 6	n = 26	n = 7	n = 6
Median (Q1–Q3)	46 (35–63)	50 (47–68)	63 (19–66)	54 (42–66)	41 (21–81)	55 (44–89)
Mean (STD)	58 (44)	54 (13)	51 (27)	55 (15)	46 (29)	59 (27)
**Zinc (mmol/L)**	n = 24	n = 11	n = 8	n = 24	n = 14	n = 19
Median (Q1–Q3)	9.6 (7.4–11.1)	9.3 (7.4–11.1)	8.5 (7.6–8.9)	8.9 (7.2–10.5)	9.8 (7.8–10.4)	8.5 (7.6–10.5)
Mean (STD)	10.3 (4.2)	9.4 (2.4)	8.4 (1.0)	10.2 (5.6)	9.3 (1.8)	9.15(2.77)

Normal Values: Selenium 46–143 µg/L; Albumin 35–52 g/L; Iron 10.3–30.9 mmol/L; Vitamin A 1.05–2.80 mmol/L;

Vitamin D 49–172 nmol/L; Zinc 8.2–23 mmol/L

**Table 6:** Child Biochemistry.

	Child Males			Child Females		
	Baseline	Visit 1	Visit 2	Baseline	Visit 1	Visit 2
Selenium (µg/L)	n = 25	n = 19	n = 16	n = 20	n = 14	n = 15
Median (Q1–Q3)	53 (48–56)	72 (63–82)	67 (62–72)	45 (40–49)	68 (58–72)	66 (56–74)
Mean (STD)	52 (7)	73 (12)	67 (7)	45 (8)	67 (9)	65 (14)
Albumin (g/L)	n = 26	n = 24	n = 24	n = 22	n = 21	n = 22
Median (Q1–Q3)	44 (43–45)	45 (43–46)	43 (41–44)	45 (43–46)	46 (43–48	43 (42–44)
Mean (STD)	44 (2)	44 (2)	43 (3)	44 (2)	45 (3)	43 (2)
Iron (mmol/L)	n = 26	n = 24	n = 24	n = 22	n = 21	n = 22
Median (Q1–Q3)	10.6 (8–14.7)	15 (12–20)	11.0 (8.4–15.6)	10.1 (7.7–15)	12.1 (6.7–14.7)	10.6 (6.4–15.1)
Mean (STD)	11.9 (5.2)	16 (6)	13.0 (8.1)	10.9 (4.1)	12.2 (7.6)	10.7 (5.0)
Vit A (mmol/L)	n = 26	n = 24	n = 23	n = 21	n = 20	n = 22
Median (Q1–Q3)	0.97 (0.73–1.07)	0.99 (0.84–1.13)	1.00 (0.89–1.08)	0.82 (0.76–1.03)	1.02 (0.89–1.22)	0.92 (0.83–1.18)
Mean (STD)	0.93 (0.26)	1.00 (0.24)	1.00 (0.19)	0.92 (0.25)	1.05 (0.22)	1.00 (0.28)
Vit D (nmol/L)	n = 27	n = 9	n = 10	n = 19	n = 10	n = 9
Median (Q1–Q3)	66 (55–73)	71 (62–75)	54 (47–73)	67 (59–79)	58 (41–60)	70 (50–73)
Mean (STD)	66 (14)	70 (12)	60 (17)	70 (20)	57 (17.4)	65 (15)
Zinc (mmol/L)	n = 20	n = 20	n = 18	n = 21	n = 15	n = 19
Median (Q1–Q3)	10.4 (9.8–10.1)	8.8 (6.8–10.1)	11.8 (10.1–14.2)	10.4 (10–11.4)	8.6 (6.2–10.5)	14.3 (11.1–16.3)
Mean (STD)	8.9 (2.0)	8.9 (1.0)	12.4 (3.2)	10.3 (2.0)	8.5 (1.9)	14.1 (3.2)

Normal Values: Selenium 46–143 µg/L; Albumin 38–54 g/L; Iron 9.0–21.5 mmol/L; Vitamin A 0.7–1.5 mmol/L;

Vitamin D 49–172 nmol/L; Zinc 8.2–23 mmol/L Increases in all micronutrients except for vitamin D (adults and children) and zinc (adults) were observed.

We fitted multiple regression models for each micronutrient to investigate the effect of supplementation accounting for sex, age, HIV status or weight. After adjusting for the effect of HIV status and weight, selenium levels increased on average by 9.66 µg/L (95% CI 4.47–14.84 p<0.01) at Visit 1, and did not increase much further at Visit 2 (10.32 µg/L, 95% CI 5.50–15.13) compared to baseline. When sex, age, and weight were adjusted for, there was a significant increase in albumin of 1.14 g/L (95% CI 0.21–2.07 p = 0.02) at Visit 1, but this benefit was lost by Visit 2. Likewise, iron increased by 1.98 mmol/L (95% CI 0.30–3.66 p = 0.02) at Visit 1, but this benefit disappeared by Visit 2 in models adjusted for sex, age and weight. Vitamin A increased significantly at Visit 1 (0.09 mmol/L, 95% CI 0.003–0.18 p = 0.04), and this increase was sustained at Visit 2 in a model adjusted for HIV status and weight. Zinc significantly decreased at Visit 1 (-1.75 mmol/L, 95% CI -2.67–-0.83 p<0.01), but returned to baseline levels by Visit 2.

## Discussion

This pilot study set out to explore the impact of a fortified supplementary food–e’Pap-on the health and well-being of crèche children and adult TB patients in Alexandra. The study was carried out with significant resource limitations amongst a deprived population group. These constraints led to the Research Team developing an open label, longitudinal design without a placebo controlled comparator group. Given that this population is believed to have poor nutritional status, it was also deemed unethical to offer a placebo supplementary food. The nature of the study population and setting made it impossible to control for all confounding variables, such as the impact of de-worming or TB-medication. Similarly, the small sample size and loss-to-follow-up have limited the statistical significance of some findings. Despite this and the reduced number of blood sample analyses eligible for inclusion, the remaining sample allowed statistical analysis which showed significant changes over time. The relatively short duration of the study did not allow inferences about the long-term impact or sustainability of this type of intervention.

As the health workers each dealt with a different indicator, consistent changes across different indicators were unlikely to be the result of bias. Furthermore, the health workers had no stake in, or expectation of, any particular outcome, and the importance of impartial recording of data was repeatedly emphasised during training and on site.

The high rates of unemployment and self-reported HIV positive status among the adult cohort are above the national averages [38], [39], but these findings are not surprising in the resource-poor setting of Alexandra and context of widespread co-infection with HIV and TB.

The improved general well-being among the adults, which was accompanied by fewer reports of low energy, was in stark contrast to the Manary study [40], which used Corn Soya Blend (CSB) and a ready-to-use peanut butter lipid paste.

The observed increase in BMI and stagnancy in waist to hip ratios among adult participants suggests that BMI increases might be due to muscle gain rather than fat gain. This was similar to results in the child cohort where improvements were observed in lean body mass–Figure 2.

In all cases, mean MUAC in centimetres was far above the cut-off value for moderate malnutrition (13.5), and it decreased over the course of the study from baseline values of 16.9 for boys and 17.2 for girls. This suggests that MUAC may not be a sensitive measurement in moderate malnutrition.

The improvement in the Illness Marker measured by bioelectrical impedance analysis could be an indication of improved cellular health and membrane integrity as a result of combined nutritional supplementation and medication among adults. The baseline values measured in both adult and child cohorts were closer to levels expected from critically ill subjects. Due to the reasonably good health that was observed among children in this study, there could be age-related differences in normal Illness Marker values. This suggests the need to conduct a survey to gather normal values, specifically for South African populations.

The BIA results for both Lean Body Mass and the Illness Marker suggest that this device could provide useful measures of change in nutritional status. However, the values determined by multi-frequency bioelectrical impedance analysis remain indicative and need to be validated in this population.

Though developmental norms for African populations are lacking, hand-grip strength improved in adult and child cohorts, supporting the view that the increases in BMI are likely to be secondary to improvements in lean muscle mass. Hand-grip strength appears to be a useful indicator of change in nutritional status.

The study population's subsistence diet relies heavily on pap (refined maize porridge) and bread, which are fortified with vitamin A, zinc, iron, folic acid and other vitamins according to SA Government guidelines [29]. However, despite dependence on these fortified foods, the data shows high levels of micronutrient deficiency at baseline, suggesting that fortification of these staples with inorganic minerals does not result in nutrient repleteness [35], [36].

The improvement in iron levels from baseline to Visit 1 is notable, as similar improvements have not been achieved after more than five years of iron fortification of wheat and maize flour in South Africa [41].

Zinc levels in children showed an initial decrease at visit 1, followed by an increase at visit 2. This may be due to an increased utilisation of these micronutrients, followed by replenishment of depleted stores, as depressed immune systems are activated as a result of improved nutrition.

## Conclusions

It should be noted that this population's subsistence diet consists primarily of foods which are fortified according to government guidelines [29]; yet very low micronutrient levels were found at baseline.

Despite the short period of intervention, there were statistically significant improvements in key micronutrient levels, well-being and energy, hand-grip strength, the BIA Illness Marker, and certain clinical indicators.

BMI and MUAC are frequently used as standard measures to evaluate the efficacy of nutritional interventions. However, weight gain does not necessarily indicate improved nutritional status. Hand-grip strength, lean body mass and the BIA Illness Marker appear promising indicators of nutritional state as they were more sensitive to change over time and they reflect functional change.

The research indicated a beneficial effect of e’Pap for both study populations, and particularly for adult TB patients, whose baseline data reflected severe food insecurity and malnutrition in a majority of cases.

Poor food security and dietary diversity undermine health and are likely to undermine conventional TB treatment and early childhood development. The improvement in serum micronutrient levels over the short time period of this study suggest that fortified supplementary foods, such as e’Pap, with bio-available micronutrients could be of great benefit, particularly if incorporated into health interventions in resource poor settings.

Though suggestive, this study’s limitations prevent firm conclusions. Wider investigation with a larger sample size, increased duration of intervention and longer term follow-up to ascertain the sustainability of the positive outcomes is recommended. A detailed and more resource-intensive follow up study could inform public health interventions aimed at addressing micro-nutrient deficiency and improving the efficacy of conventional treatment protocols.
